# Herpesvirus Late Gene Expression: A Viral-Specific Pre-initiation Complex Is Key

**DOI:** 10.3389/fmicb.2016.00869

**Published:** 2016-06-06

**Authors:** Henri Gruffat, Roberta Marchione, Evelyne Manet

**Affiliations:** ^1^International Center for Infectiology Research, Oncogenic Herpesviruses Team, Université de Lyon, LyonFrance; ^2^Inserm, U1111, LyonFrance.; ^3^Ecole Normale Supérieure de Lyon, LyonFrance; ^4^CNRS, UMR5308, LyonFrance; ^5^Université Lyon 1, LyonFrance

**Keywords:** herpesviruses, Epstein-Barr virus, PIC, late gene, TBP-like, viral gene expression

## Abstract

During their productive cycle, herpesviruses exhibit a strictly regulated temporal cascade of gene expression that can be divided into three general stages: immediate-early (IE), early (E), and late (L). This expression program is the result of a complex interplay between viral and cellular factors at both the transcriptional and post-transcriptional levels, as well as structural differences within the promoter architecture for each of the three gene classes. Since the cellular enzyme RNA polymerase II (RNAP-II) is responsible for the transcription of herpesvirus genes, most viral promoters contain DNA motifs that are common with those of cellular genes, although promoter complexity decreases from immediate-early to late genes. Immediate-early and early promoters contain numerous cellular and viral *cis*-regulating sequences upstream of a TATA box, whereas late promoters differ significantly in that they lack *cis*-acting sequences upstream of the transcription start site (TSS). Moreover, in the case of the β- and γ-herpesviruses, a TATT box motif is frequently found in the position where the consensus TATA box of eukaryotic promoters usually localizes. The mechanisms of transcriptional regulation of the late viral gene promoters appear to be different between α-herpesviruses and the two other herpesvirus subfamilies (β and γ). In this review, we will compare the mechanisms of late gene transcriptional regulation between HSV-1, for which the viral IE transcription factors – especially ICP4 – play an essential role, and the two other subfamilies of herpesviruses, with a particular emphasis on EBV, which has recently been found to code for its own specific TATT-binding protein.

## Introduction

Herpesviridae form a large family of enveloped double-stranded DNA viruses with large and complex genomes ranging from 120 to 250 kbp in length. Herpesviruses have long co-evolutionary histories with their natural hosts. The consequences of this co-evolution are a wide virus incidence in the natural host populations and infections leading to virus persistence in specific cell types. Occasional reactivation from infected cells allows these viruses to maintain a dynamic infectious reservoir for transmission of infectious virions to new naïve hosts. Herpesviruses are categorized into three sub-families according to their cellular tropism, pathogenicity, and behavior under culture conditions in the laboratory. α-herpesviruses establish latency in neurons; they have a rapid replication cycle and can infect a broad variety of species in experimental animal systems as well as a wide range of cells *in vitro*. β-herpesviruses establish latency in macrophages and lymphocytes and γ-herpesviruses in lymphocytes. Both β- and γ-herpesviruses replicate more slowly and in a more restricted range of cells than α-herpesviruses. Members of each of the three sub-families of herpesviruses have been described to cause characteristic diseases in humans (**Table 1**). The ability of herpesviruses to establish a latent state of infection with low levels of viral gene expression, for the life of the host, is a defining feature of these pathogens.

**Table 1:** Human herpesvirus (HHV) classification.

Subfamily	Name	Synonym	Genome length	Primary target	cell	Pathophysiology	Site of latency	Means of spread
**α**	HHV-1	Herpes simplex virus-1 (HSV-1)	135 472 bp	Mucoepithelial	Oral and genital herpes, as well as other herpes simplex infections (gingivostomatitis, keratitis, and dermal whitlows, encephalitis).	Neuron	Close contact (oral or sexually transmitted infection)
	HHV-2	Herpes simplex virus-2 (HSV-2)	154 746 bp	Mucoepithelial		Neuron	Close contact (oral or sexually transmitted disease)
	HHV-3	Varicella zoster virus (VZV)	124 884 bp	Mucoepithelial	Chickenpox and shingles	Neuron	Respiratory and close contact
**β**	HHV-5	Cytomegalovirus (hCMV)	229 354 bp	Monocyte, lymphocyte, and epithelial cells	Congenital cytomegalovirus infection (when symptomatic) causes hepatosplenomegaly, retinitis, rash, and central nervous system involvement. Infectious mononucleosis-like syndrome. Immunocompromised hosts may develop life-threatening disseminated disease involving the lungs, gastrointestinal tract, liver, retina, and central nervous system.	Monocyte, lymphocyte	Saliva, urine, milk
	HHV-6A HHV-6B	Roseolovirus, Herpes lymphotropic virus	159 321 bp	T cells	Associated with exanthem subitem (roseola) and with rejection of transplanted kidneys.	T cells	Close contact (saliva) or respiratory
	HHV-7		144 861 bp	T cells		T cells	
**γ**	HHV-4	Epstein-Barr virus (EBV)	171 823 bp	B cells and epithelial cells	Causative agent for Infectious mononucleosis. Associated with: Burkitt’s lymphoma, Hodgkin diseases post-transplant lymphoproliferative syndrome, nasopharyngeal carcinoma, a subtype of gastric carcinoma.	B cells	Close contact (saliva), transfusions, tissue transplant
	HHV-8	Kaposi’s sarcoma-associated herpesvirus (KSHV)	138 146 bp	Lymphocyte and other cells	Kaposi’s sarcoma, primary effusion lymphoma, some types of multicentric Castleman’s disease	B cells	Close contact (sexual), saliva

**FIGURE 1:**
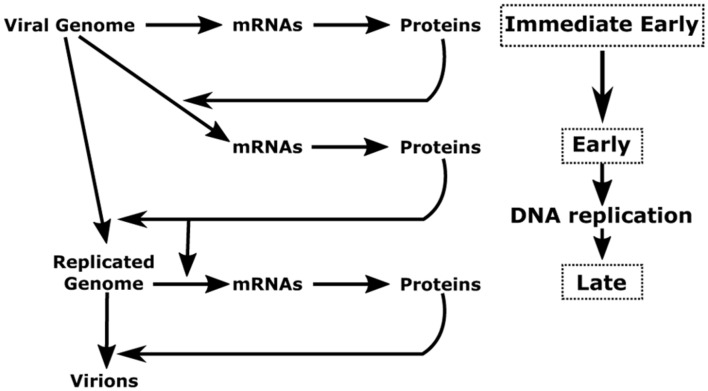
**Schematic representation of herpesvirus productive cycle.** The productive cycle begins with the expression of the immediate-early genes: The immediate early genes present on the viral genome are transcribed to mRNA and then translated into proteins. These products are transcription factors required for activation of the early genes. These genes are also transcribed from the viral genome. The products of early genes are involved in (i) the formation of the viral DNA replication complex, (ii) the composition of the viral transcription complex responsible for expression of the β- and γ-herpesvirus late viral genes, (iii) the cytoplasmic accumulation of a subset of early and late viral mRNAs. Following amplification of the viral genome by the viral DNA replication complex, the late viral genes are expressed probably from the newly replicated viral DNA. Most of the late gene products contribute to the formation of the viral particle. The four stages (immediate-early, early, DNA replication and late) of the viral productive cycle are indicated in the right part of the scheme. The detailed steps of the productive cycle are indicated in the left part of the scheme.

The herpesvirus cycle is characterized by two distinct phases: latency and the productive or lytic cycle. During latency, no active viral production occurs and a very limited number of viral genes are expressed. Under appropriate stimuli, the herpesvirus productive cycle is activated leading to the genesis of new virions. A temporally regulated cascade of gene expression – during which three distinct kinetic classes of transcripts are expressed in a sequential manner – characterizes the viral productive cycle (**Figure 1**). It should be noted that the great majority of viral genes are expressed during the productive cycle and that all protein-coding genes are transcribed by cellular RNA polymerase II (RNAP-II) (Costanzo et al., 1977).

The productive cycle starts with the expression of the immediate-early (also refers to as IE or α) genes characterized by their ability to be transcribed in the absence of *de novo* protein synthesis. These genes encode for viral regulatory proteins involved in transcriptional control of the early (also refers to as E or β) genes. The products of the viral early genes are required for (i) the subsequent replication of the viral genome; (ii) the expression of the late (also refers to as L or γ) viral genes; and (iii) the cytoplasmic accumulation of some of the early and late viral mRNAs. For viral DNA amplification, herpesviruses encode their own DNA replication machinery as well as a number of enzymes that contribute to the biosynthesis of deoxynucleoside triphosphates. In addition, it has been shown in the case of the α-herpesvirus, HSV-1, that the function of several early genes is to turn off the synthesis of the immediate early gene products and to induce the expression of the late genes (Honess and Roizman, 1974, 1975). In general, expression of HSV-1 early genes is not dependent on viral DNA replication. However, the continued accumulation of a subset of early genes – called early late or β2 – is enhanced by the onset of viral DNA replication.

Following viral DNA replication, late (L or γ) viral genes – that mainly encode structural proteins – start to be transcribed, ultimately leading to the assembly and release of infectious particles. The late genes are subdivided into two classes (γ1 and γ2) based on their apparent requirement for DNA replication. Expression of the γ1 genes is delayed compared to that of the early genes. By contrast, expression of the γ2 genes appears to be strictly dependent upon the onset or completion of lytic DNA amplification. Their expression is neither observed in the presence of inhibitory concentrations of drugs that block viral DNA synthesis nor with viruses carrying conditional mutations in some of the early genes at the non-permissive temperature.

How the immediate-early and early genes are regulated is now quite well defined, whereas the mechanisms involved in transcriptional regulation of the late viral genes have been much less characterized. A central question concerning the regulation of viral gene expression is the nature of *cis*-acting sequences and viral *trans*-acting signals, which permit the cellular transcriptional machinery to differentiate between immediate early, early, and late genes. Moreover, although it has been long known that late viral gene transcription is tightly coupled to viral DNA replication, the underlying mechanism is poorly understood, especially for the β- and γ-herpesviruses. This review will focus on recent studies that have started to unravel some of the mechanisms involved in β- and γ-herpesviruses late gene transcription.

## Dependence Upon Viral DNA Replication

For all DNA viruses, it is well known that viral gene expression is temporally regulated during productive infection and is generally divided into early and late phases separated by viral genome replication. For the herpesviruses, according to the model initially based on the study of HSV-1, late gene expression is strictly dependent on the lytic viral DNA replication (Honess and Roizman, 1974; Stinski, 1978; Mavromara-Nazos and Roizman, 1987). An identical model has been proposed for EBV and MHV-68 on the basis of experiments using inhibitors of the viral DNA polymerase, such as phosphonoacetic acid (PAA; Summers and Klein, 1976; Datta and Hood, 1981; Martinez-Guzman et al., 2003). Treatment of EBV-infected cells by PAA not only blocks viral lytic DNA replication, but also expression of the late EBV genes (Serio et al., 1997). This model is supported by the observation that the Raji cell line – which carries a mutated EBV genome with a deletion in the single-stranded DNA-binding protein gene – is defective for both lytic viral DNA replication and late gene expression. Thus, viral DNA replication appears to constitute an important temporal boundary between early and late gene expression in all herpesviruses.

Interestingly, when HSV-1 late viral promoters are removed from their viral context and introduced into either plasmid vectors or the host genome, they no longer display the characteristics of late viral promoters (Dennis and Smiley, 1984; Silver and Roizman, 1985; Wade et al., 1992; Wade and Spector, 1994; Chang and Ganem, 2000). One possible explanation for the need for viral DNA replication in the viral context, is that non-replicated viral genomes cannot serve as templates for late gene expression, even if all viral *trans*-acting factors are expressed via a separate viral genome replicated *in trans* (Mavromara-Nazos and Roizman, 1987). This hypothesis is supported by the fact that once a lytic origin of replication is provided *in cis* to a late promoter on a plasmid vector, proper late gene regulation can be observed in infected cells (Johnson and Everett, 1986a; Amon et al., 2004). Taken together, these experiments suggest that the induction of the HSV-1 γ2 genes is mediated by a *cis*-acting function associated with viral DNA synthesis. Thus the viral origin of DNA replication (OriLyt) is a critical *cis*-acting element regulating replication-dependent induction of herpesvirus late promoters.

## *Cis*-Acting Elements

**FIGURE 2:**
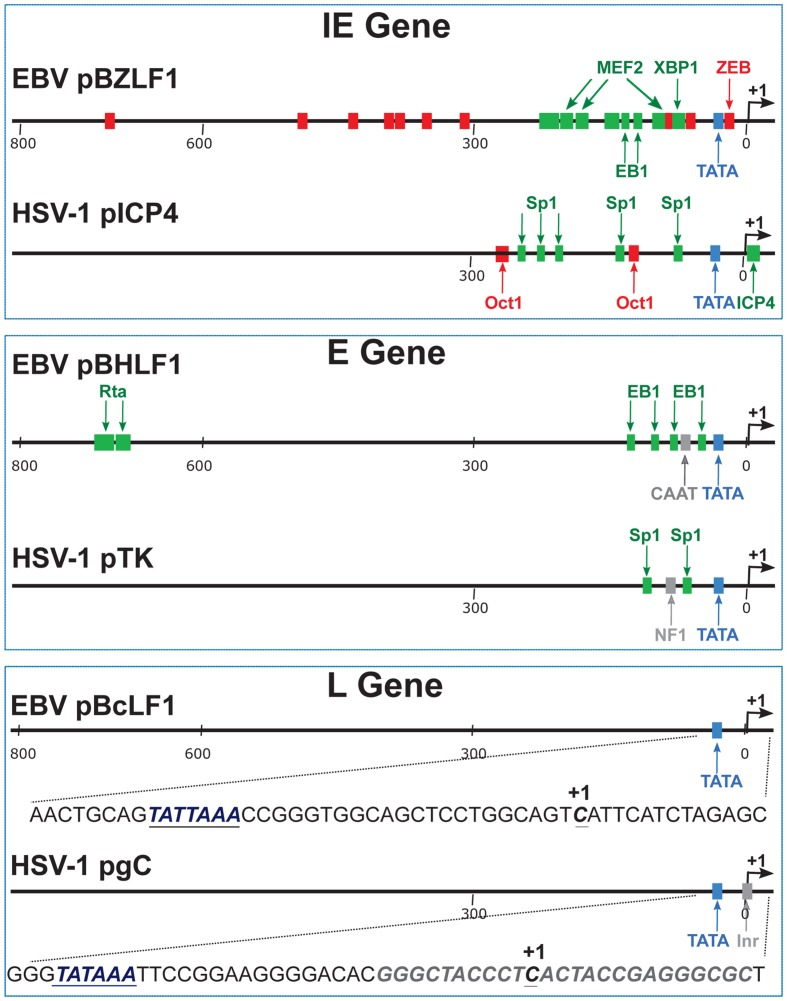
**Schematic representation of typical immediate-early, early and late gene promoters.** These examples come from EBV and HSV-1. pBZLF1 is the promoter of the EBV immediate-early gene encoding the viral transcription factor called EB1, BZLF1, or Zta. pICP4 is the promoter of the HSV-1 immediate-early gene encoding the viral transcription factor called ICP4. The negative *cis*-regulatory elements are indicated in red. The positive *cis*-regulatory elements are labeled in green. pBHRF1 is the promoter of the EBV early gene encoding the BHRF1 protein. EB1 and R are the viral transcription factors responsible for the activation of early gene expression. pTK is the promoter of the HSV-1 early gene encoding the Thymidine Kinase protein. pBcLF1 is the promoter of the EBV late BcLF1 gene that encodes the major capsid protein. pgC is the promoter of the HSV-1 late gene encoding the glycoprotein C. The viral late promoters do not contain any positive or negative *cis*-regulatory elements. In addition to the TATA box element, HSV-1 late promoters contain an initiator element (inr). By contrast to HSV-1, EBV late promoters contain a TATT box at the place of the canonical TATA box. IE, immediate early; E, early; L, late.

Viral gene expression is largely regulated at the transcriptional level, genes being transcribed by cellular RNAP-II (Godowski and Knipe, 1986; Weinheimer and McKnight, 1987). In order to define the *cis*-acting elements required for the expression of late viral genes, the promoter regions of several late genes from different herpesviruses were cloned upstream of a reporter gene. The activity of these promoters – or sets of promoter deletion mutants – was then assessed following transfection of the various constructs into infected cells. Up to now, few late viral promoters have been studied in such detail, but in all cases examined the same type of simplistic promoter structure has been revealed: a sequence comprised between positions -30 and +30 relative to the transcription start site (TSS), including a TATA box motif, appears to be the only important sequence allowing typical late gene regulation of transcription (Johnson and Everett, 1986b; Shapira et al., 1987; Chang et al., 1998; Serio et al., 1998; Tang et al., 2004). This absence of *cis*-acting elements in late viral promoters is in sharp contrast with what is known for cellular or viral immediate-early and early gene promoters (**Figure 2**).

In the case of the HSV-1 *gC* late gene, a study by Homa et al. (1988) identified a 15 bp sequence surrounding the TATA box as being sufficient for this particular late viral gene’s transcription. For the sake of simplicity, such a minimal sequence, including a TATA box motif, will be hereafter referred to as the TATA element. Interestingly, replacement of the gC sequence by the TATA element from an early viral gene promoter resulted in a transcriptionally inactive *gC* gene. However, if the substitution was made using another late gene TATA element, the *gC* gene was transcribed normally.

Comparison of TATA elements from several early and late HSV-1 genes did not reveal any obvious differences to account for one sequence functioning as a late promoter and another not. A chimeric HSV-1 gene construct that contained the distal regulatory elements of the early TK gene, fused upstream of the TATA element from the late *gC* gene, exhibited both early and late kinetics of gene expression. This study indicates that late gene expression requires the presence of a TATA element in the promoter but no further upstream sequences, whereas *cis*-acting regulatory elements are required for early gene expression (Homa et al., 1988).

An interesting case concerns the hCMV UL44 gene that encodes the double-stranded DNA-binding protein. The UL44 transcription unit initiates at three distinct sites that are separated by around 50 nucleotides and are differentially regulated during productive infection. Two of these start sites (the distal and proximal) are used at early times, whereas the middle start site is used only at late times. Moreover, expression from the late start site is dependent upon viral DNA synthesis. Interestingly, the UL44 early viral promoters have canonical TATA sequences (TATAA), whereas the late viral promoter displays a non-canonical TATA sequence (TATTATTA). Mutations in this TATTATTA motif, significantly affect the level of late viral gene expression, attesting to the importance of the motif (Chang et al., 1989; Leach and Mocarski, 1989; Isomura et al., 2007). Moreover, replacement of the distal canonical TATA box by the non-canonical TATTATTA sequence induced the expression of an alternative transcript at late times after infection, indicating that the non-canonical TATA box directs expression of the gene at late times of infection (Isomura et al., 2008).

**FIGURE 3:**
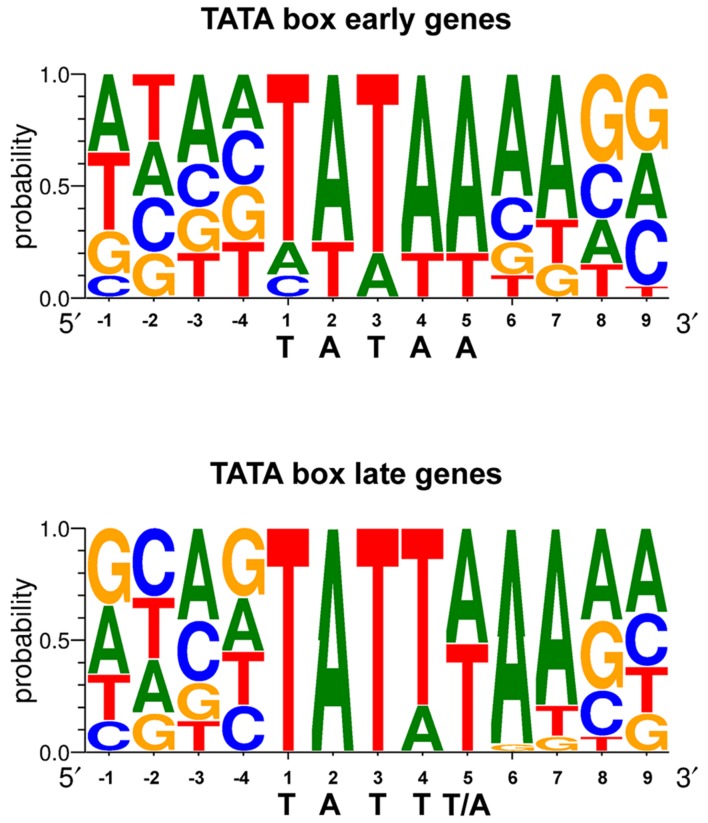
**TATA box sequence conservation of early and late EBV genes presented by sequence logo.** For each position, the percentage of occurrence of each DNA base is indicated.

The region responsible for conferring late gene regulation in the case of the EBV BcLF1 gene (encoding the major capsid protein), also contains a TATA box variant (TATTAAA; Serio et al., 1998). The presence of this type of non-canonical TATA box sequence in EBV late promoters appears to be one of the determinants of late promoter activity and most of the EBV late promoters display this unusual TATT motif (**Figure 3**). This also appears to be the case for KSHV and MHV68 (Wong-Ho et al., 2014). Replacement of the T at the fourth position of the TATT box by an A, led to a complete loss of late gene expression specificity (Serio et al., 1998; Gruffat et al., 2012), although this is not absolute (Amon et al., 2004). It is interesting to note that some of the early genes also contain a non-canonical TATT box, suggesting that the sequence of the box by itself is not sufficient to determine the timing of the expression of the gene.

Thus, the TATA box - or its TATT variant - appears to be the major control element required to determine the transcription initiation site, the strength of transcription and the timing of expression, of the late viral genes.

## *Trans*-Acting Elements

From recent data, it is clear that a strong difference exists between α-herpesviruses and β- or γ-herpesviruses in terms of the viral proteins required for the expression of the late viral genes. This difference may reflect the variation observed in the type of TATA-box sequence used to control the expression of the late genes: a TATA-box for the α-herpesviruses vs. a TATT-box for the β- and γ-herpesviruses. Transcriptional activation of α-herpesvirus late genes requires immediate-early proteins, especially the ICP4 transcription factor. By contrast, the β- and γ-herpesvirus immediate-early proteins are dispensable for late gene expression, but these viruses encode specific early proteins that *trans*-activate the late genes through the TATT-box.

## α-Herpesviruses

Transcription initiation is a major control point for both viral and cellular gene expression and requires the proper assembly of the general cellular transcription factors TFIIA, TFIIB, TFIID, TFIIE, TFIIF, and TFIIH, as well as RNAP-II to form pre-initiation complexes (PIC) on core promoter elements. PIC formation begins with the binding of the general transcription factor TFIID, a multi-subunit complex composed of the TATA-binding protein (TBP) together with around 14 TBP-associated factors (TAFs). Although TBP is sufficient for basal transcription initiation, activated transcription requires the TAFs (Buratowski, 1994). Many cellular and viral transcription factors have been shown to interact with the general transcription machinery. TAFs also play an important role in the recognition of non-TATA box core promoter elements such as INRs (initiator elements) that can be present in place of – or in addition to – a TATA box on core promoters (Juven-Gershon et al., 2008). TFIID binding to the TATA box and/or to an INR is stabilized by TFIIA and TFIIB. Whereas TFIIB is essential for basal transcription directed by either TBP or TFIID, TFIIA is dispensable for TBP-directed basal transcription, although it can stabilize TBP interaction with the TATA box (Lee et al., 1992). The formation of a TFIID-TFIIA-TFIIB-DNA complex allows the subsequent recruitment of the other general transcription factors, TFIIE, TFIIF, TFIIH, and RNAP-II. These are recruited individually, or as preformed complexes, to the core promoters, completing the PIC assembly, which is then ready to initiate transcription. The recruitment of TFIID and the general transcription factors is crucial for the regulation of both early and late viral gene transcription. Hence, the efficiency of the formation of the PIC on the viral promoters is a critical step in determining the rate of transcription. Promoter recognition and binding by TFIID, as well as PIC formation, are enhanced by the action of activator proteins. One well-studied viral trans-activator, the VP16 protein from HSV-1, activates viral immediate-early gene expression. Its acidic activation domain is assumed to enhance the formation of the PIC by interacting with TFIIB. Thus, VP16 does not facilitate TFIID binding to the TATA box, but rather enhances TFIIB binding to the complex, which then promotes RNAP-II recruitment. In addition, VP16 interacts with TFIIA and this interaction also facilitates PIC assembly (Kobayashi et al., 1995, 1998).

**FIGURE 4:**
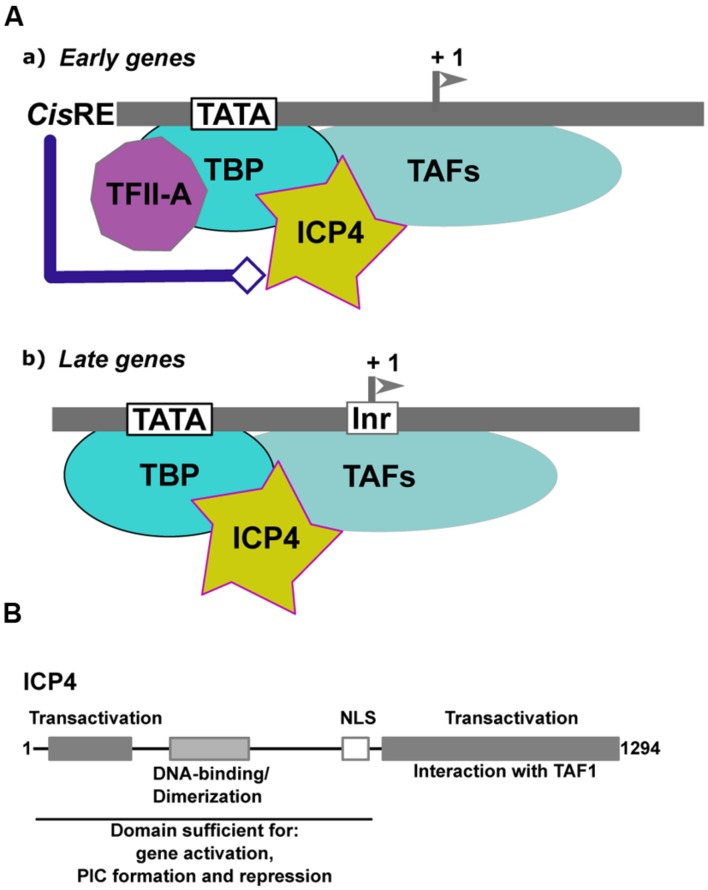
**Transcriptional regulation of α-herpesviruses late gene expression. (A)** Schematic representation of the initial step of formation of the Pre-Initiation Complex (PIC) on early and late α-herpesvirus promoters. (a) Early promoters contain either a TATA box, an initiator element (Inr) or both, associated with different *cis-*regulatory elements (*cis-*RE). TFIID (TBP associated with the TAFs) is stabilized on the promoter via its interaction with TFIIA. The structure of the viral chromatin is probably modified by the *cis*-regulatory factors to allow formation of the PIC and recruitment of ICP4. (b) Late promoters are composed of a TATA box associated with an Inr. The expression level of TFIIA is reduced within cells during the productive cycle. Recruitment of TFIID onto the late viral promoters is stabilized by ICP4. **(B)** Schematic representation of ICP4 functional domains. Gray boxes and text describe the various domains of ICP4 and their function. The functional domains of ICP4 include two transcriptional regulatory regions localized in the N- and C-terminal parts of the protein, respectively, separated by a region carrying a dimerization domain, a DNA-binding domain with a helix-turn-helix motif, and a nuclear localization sequence (NLS).

Infected-cell polypeptide 4 (ICP4) is one of the five IE proteins of HSV-1. ICP4 (**Figure 4A**) is a regulator of viral transcription required for the efficient transcription of both early and late viral genes (Watson and Clements, 1980; Everett, 1984; DeLuca and Schaffer, 1985; O’Hare and Hayward, 1985a; Godowski and Knipe, 1986; Sampath and DeLuca, 2007). ICP4 increases the transcription rates of several viral genes (Godowski and Knipe, 1986), but also represses the transcription of certain others (Everett, 1984; DeLuca and Schaffer, 1985; O’Hare and Hayward, 1985a,b; Godowski and Knipe, 1986). ICP4 does not appear to require any specific binding sites to activate transcription, (Everett, 1984; Coen et al., 1986). By contrast, the binding of ICP4 to specific sites has been shown to be involved in repression, as in the case of the LAT gene promoter (Freeman and Powell, 1982; Faber and Wilcox, 1986; Roberts et al., 1988; Gu et al., 1995).

In the case of HSV-1, initiator elements (INRs) which overlap the initiation start site, have been found to be important, in addition to the TATA box, for late gene regulation (Steffy and Weir, 1991; Guzowski and Wagner, 1993; Guzowski et al., 1994). INRs are common to many cellular core promoters and can promote transcription initiation in the absence of a TATA box. However, there are also examples where INRs can synergize with a TATA box (reviewed in reference Smale and Kadonaga, 2003). These elements are specifically recognized by components of the general transcription factor TFIID (Kaufmann et al., 1998). The INR is the only necessary element, in addition to the TATA box, for efficient activation of the late promoters by ICP4. In an *in vitro*-reconstituted transcription system, ICP4 absolutely requires *cis-*acting factors to activate transcription from a representative early promoter. In the case of the late viral promoters, ICP4 appears to stabilize the formation of the PIC via enhancement of TFIID’s binding to the promoter (Carrozza and DeLuca, 1996; Grondin and DeLuca, 2000). Moreover, there is a differential requirement for general transcription factors between the early and late gene promoters: TFIIA is required for efficient ICP4 activation of the early TK promoter but is dispensable for ICP4 activation of the late *gC* promoter (Gu and DeLuca, 1994; Carrozza and DeLuca, 1998; Zabierowski and DeLuca, 2004). The dispensability of TFIIA for activation of this promoter by ICP4 is dependent on an intact INR. Interestingly, it has been shown that during the infection process and the viral productive cycle, the expression of TFIIA decreases dramatically (Zabierowski and DeLuca, 2004). Taken together, these observations suggest that, in the case of the regulation of late viral gene expression, ICP4 can substitute for TFIIA by stabilizing TFIID, but only when the promoter contains a functional INR. In the case of early promoters, ICP4 facilitates the ability of TFIIA to stabilize the binding of TBP to the TATA box.

Moreover, ICP4 co-purifies with a subset of TAFs (TAF-3, TAF-4, TAF-5, TAF-6, and TAF-9), suggesting that ICP4 can stably associate with different types of the TFIID complex formed at different times post-infection on the early and late viral genes in infected cells (Lester and DeLuca, 2011). The TAF-1 amino-terminal domain contains a negative regulatory element that inhibits TBP-TATA complex formation. TFIIA has been shown to competitively de-repress the inhibitory effect of TAF-1 by destabilizing the interactions between TAF-1 and TBP (Ozer et al., 1998). By interacting with TAF-1, ICP4 may function in a manner analogous to TFIIA in that it may alleviate the inhibitory effects of TAF-1 (**Figure 4A**). Two transactivation domains that cooperate to activate viral gene expression have been mapped in ICP4, one in the N-terminal and the other in the C-terminal of the protein (**Figure 4B**). A mutant of ICP4 lacking the C-terminal activation domain efficiently activates many early genes, whereas late genes are poorly activated and virus growth is severely impaired (DeLuca and Schaffer, 1987; Wagner et al., 2012b). Interestingly, the N-terminal regulatory domain of ICP4 is involved in the ICP4-TBP interaction (Wagner et al., 2012a), while interaction with TAF-1 is dependent on an intact ICP4 C-terminal domain (Carrozza and DeLuca, 1996; Brown et al., 1999; Wagner et al., 2012a). The N-terminal region of ICP4 interacts with TBP to stabilize its interaction with the TATA box in the context of TFIID. This may explain why ICP4 differentially affects early and late promoter regulation (**Figure 4B**).

In addition to ICP4, it has been shown that regulation of the late viral gene, gC, requires the viral ICP27 protein (Jean et al., 2001). ICP27 is an immediate-early protein essential for virus production. It is conserved among all herpesviruses and is implicated in the cytoplasmic accumulation of some early and late viral mRNAs transcribed from intronless genes (Sandri-Goldin and Mendoza, 1992; Juillard et al., 2012). However, the ability of ICP27 to promote late gene expression appears to be distinct from its function in mRNA export. In this case, the effect is apparently at the mRNA transcription level and could be linked to the finding that ICP27 interacts directly with the C-terminal domain of RNAP-II.

In addition to its role in viral DNA amplification, the viral early protein ICP8 – a single stranded DNA-binding protein – is thought to play a role in viral late gene expression. In effect, in cells expressing a dominant negative form of ICP8, both late protein synthesis and the accumulation of late gC mRNA are reduced during HSV-1 infection (Godowski and Knipe, 1986; Gao and Knipe, 1991; Chen and Knipe, 1996). However, it is difficult to conclude since late gene expression is dependent on viral DNA replication.

Viral DNA replication *per se* is known to be important for late viral gene expression. However, more direct contribution by the replication complex in late gene transcription, could be envisaged. In this context, an interaction between ICP8 – which is part of the viral DNA replication complex – and RNAP-II has been reported (Zhou and Knipe, 2002; Olesky et al., 2005). This interaction requires ICP27, ICP22, UL13, and US3 kinase (Bastian and Rice, 2009).

## β- and γ-Herpesviruses

The question of how β- and γ-herpesvirus late gene expression is regulated remained unsolved for a long time, until the group of R. Sun identified several viral proteins from MHV68 (Murine HerpesVirus-68) that were required for late gene expression. Using a library of signature-tagged mutant MHV-68 viruses, Sun’s group identified 41 viral genes essential for completion of the virus life cycle (Song et al., 2005). Among these, were identified five viral genes (ORF18, ORF24, ORF30, ORF31, and ORF34) whose deletion resulted in a loss of late viral gene transcription, but with no effect on either early viral gene expression or viral DNA-replication (Jia et al., 2004; Arumugaswami et al., 2006; Wu et al., 2009; Gong et al., 2014). However, nothing was known about the function of these proteins, nor the way in which they could potentially cooperate to direct late gene expression. In particular, whether these proteins could interact with late gene promoters was unknown. Similar studies carried out with the β-herpesvirus, hCMV, showed that UL79, UL87, UL91, UL92, and UL95 (the analogs of MHV68 ORF18, ORF24, ORF30, ORF31, and ORF34, respectively) are also essential for late gene transcription (Isomura et al., 2011; Chapa et al., 2013; Omoto and Mocarski, 2013, 2014). EBV also encodes analogs of these MHV68 and hCMV proteins: BVLF1, BcRF1, BDLF3.5, BDLF4, and BGLF3, respectively. Interestingly, these genes, classified as early genes and well conserved in both the β- and γ-herpesviruses do not appear to have analogs in α-herpesviruses. A comparative survey of the productive cycle genes of various herpesviruses revealed that only seven of the viral genes that are conserved in both the β- (hCMV and HHV6) and γ- (EBV, KSHV, and MHV68) herpesvirus subfamilies are absent from HSV-1 (**Table 2**). Hence, two additional proteins – called BTRF1 and BFRF2 in the case of EBV – could potentially be involved in the specific β- and γ-herpesvirus late gene expression. However, the analog of BTRF1 in MHV68 (ORF23) was previously shown to be the product of a late gene and dispensable for virus production, both in cell culture and *in vivo*. By contrast, we recently demonstrated that BFRF2 is absolutely required for EBV late gene expression (Aubry et al., 2014). In addition, we showed that the six EBV early gene products – BVLF1, BcRF1, BDLF3.5, BDLF4, BGLF3, and BFRF2 – form a complex necessary and sufficient for the activation of a late gene reporter in EBV-negative cells.

**Table 2:** Homologous genes involved in late gene expression between beta and gamma-herpesviruses.

	γ-herpesviruses			β-herpesviruses	
Virus	EBV	KSHV	MHV68	CMV	HHV6
Gene name	BcRF1 (TBP-like)	ORF24	ORF24	UL87	U58
	BFRF2	ORF66	ORF66	UL49	U33
	BGLF3	ORF34	ORF34	UL95	U67
	BVLF1	ORF18	ORF18	UL79	U52
	BDLF4	ORF31	ORF31	UL92	U63
	BDLF3.5	ORF30	ORF30	UL91	U62

Interestingly, an *in silico* study identified structural homology between one of these genes, BcRF1, and TBP from the thermophilic archaea *Pyrococcus woesei*. Similar structural homology could also be identified with the analog hCMV protein, UL87. Although overall sequence identity is very low (around 10%), a saddle-like fold characteristic of the TBP structure as well as the presence of important residues known to be involved in the-protein-DNA interface, mark both proteins as being distantly related to TBP (Wyrwicz and Rychlewski, 2007). Accordingly, BcRF1 specifically interacts with the TATT sequence present on EBV late gene promoters (Gruffat et al., 2012). In addition to the EBV BcRF1 protein, the MHV-68 analog, ORF24, has recently been shown to specifically interact with the TATT box of MHV-68 late gene promoters (Wong-Ho et al., 2014), thus confirming the DNA-binding properties of this family of herpesvirus proteins. To date, few TBP-like proteins have been identified in eukaryotic cells, and BcRF1 and its analogs in other β- and γ-herpesviruses are the first TBP-like proteins to have been characterized from a virus, with the exception of giant viruses.

In addition to the discovery of a role for BcRF1 and its analogs, as TATT-binding proteins, a putative function has been assigned to the hCMV UL79 protein – the EBV BVLF1 analog – as a transcription elongation factor not required for the recruitment of RNAP-II onto the viral late promoters (Perng et al., 2014). UL79 co-purifies with UL87, UL95, UL49, and the hCMV DNA polymerase proteins UL112/113. In the absence of UL79, RNAP-II remains on the promoter and accumulates because it does not elongate RNA efficiently. The carboxy-terminal domain (CTD) of RNAP-II is composed of 52 repeats of the heptapeptide Y-S-P-T-S-P-S, whose phosphorylation is tightly regulated. RNAP-II is recruited unphosphorylated onto the promoter by the PIC, then released from the PIC following phosphorylation of S5 and S7 by cdk7 (TFIIH). During transcription elongation, RNAP-II CTD is fully phosphorylated whereas at the termination step, only S2 remains phosphorylated. Finally, RNAP-II dissociates from the DNA template in an unphosphorylated form (Eick and Geyer, 2013). UL79 is not involved in the phosphorylation of RNAP-II CTD at the S2 or S5 positions, suggesting that pUL79 is essential for a transcription step downstream of the initiation of elongation and before its termination, probably destabilizing a negative elongation factor.

Except for the two factors mentioned above, no function has yet been assigned to the other proteins involved in late viral transcription. However, we now know that none of these proteins is able to activate the expression of a late reporter gene in a transient transfection assay, but they can activate when they are expressed together. Furthermore, they appear to interact together to form a complex (Aubry et al., 2014; Watanabe et al., 2015). In addition, at least EBV BcRF1, MHV-68 ORF30 and ORF34, and hCMV UL95 and UL91 and KSHV ORF24 are critical for recruiting RNAP-II to the late viral promoters (Wu et al., 2009; Aubry et al., 2014; Davis et al., 2015). The formation of a large protein complex containing a TBP-like protein and able to recruit RNAP-II onto the core promoters of the late viral genes strongly suggests that the ß- and γ-herpesviruses encode for their own PIC responsible for the induction of late genes. By analogy with the cellular PIC formed around TBP, we have proposed to call this complex vPIC for viral PIC.

As discussed above, the full set of the six EBV proteins is required in order to induce the expression of a reporter gene under the control of a late promoter (Aubry et al., 2014). Interestingly, although the proteins from the different β- and γ-herpesviruses are well conserved, so far it has not been possible to exchange a particular EBV protein for its hCMV counterpart (Aubry et al., 2014). However, this appears to be possible between human and mouse CMV proteins, presumably because the two viruses are very close to each other in terms of evolution (Chapa et al., 2013). Nevertheless, the hCMV complex as a whole can induce EBV late gene expression (Aubry et al., 2014). This suggests that although the components of the complex have evolved differently, there is a strong functional conservation of the vPIC between the different β- and γ-herpesviruses.

How vPIC regulates late viral gene expression is not clear, but BcRF1 has been shown to interact with the TATT element of the viral late promoters in order to initiate transcription (Gruffat et al., 2012). This suggests that BcRF1 is at the origin of the complex’s formation on the viral late promoters. However, *in vitro*, BcRF1 binds in an undifferentiated way to both TATA and TATT motifs. Thus, how this protein discriminates between early and late promoters *in vivo* is not yet understood. One possible explanation could be that the association of BcRF1 with other components of the vPIC may favor a specific recognition of late gene promoters. In this context, the BFRF2 protein could play such a role. BFRF2 consists of two domains: a variable N-terminal part and a C-terminus well-conserved between β- and γ-herpesviruses. This latter domain contains several conserved cysteine residues, which could potentially form a zinc-finger protein. Although the detailed activity of BFRF2 needs to be further explored, it is possible that this protein could be directly involved in binding to DNA.

The six viral proteins required for the formation of the vPIC represent the minimal set of viral proteins necessary for the activation of the late viral genes. Additional accessory viral factors and cellular proteins might be required for the complete formation of vPIC, similarly to what is known for the viral DNA replication complex. In effect, herpesviruses encode seven proteins absolutely required for viral DNA replication (**Table 3**; Fixman et al., 1992, 1995), but additional accessory proteins are necessary to obtain a fully efficient replication complex. Accordingly, it has recently been shown that the EBV protein kinase, BGLF4 – analogous to KSHV and MHV68 ORF36 and hCMV UL97 proteins – which localizes to the replication compartments, participates in late gene transcriptional regulation. Abolishing expression of BGLF4 had no significant effect on either early gene expression or viral DNA replication, but significantly reduced the amount of virus released (Gershburg et al., 2007; Feederle et al., 2009; El-Guindy et al., 2014). Moreover, analysis of the EBV transcriptome revealed that the expression of 31 late genes was reduced in the absence of BGLF4. How BGLF4 controls late gene expression is however, unknown. No direct interaction between BGLF4 and vPIC components has as yet been documented, but it has been shown that the kinase activity of BGLF4 is necessary for optimal expression of the late genes, suggesting that post-translational modifications of some of the vPIC proteins could be important for their activity.

**Table 3:** Herpesviruses replication proteins.

Virus	HSV-1	EBV	CMV	Function
Gene name	UL30	BALF5	UL54	DNA polymerase (POL)
	UL42	BMRF1	UL44	Polymerase processivity factor (PPF)
	UL29	BALF2	UL57	Single-stranded DNA-binding protein (SSB)
	UL5	BBLF4	UL105	Helicase (HEL)
	UL52	BSLF1	UL70	Primase (PRI)
	UL8	BBLF2/3	UL102	Primase associated factor (PAF)
	UL9*	BZLF1*	–	Origin-binding protein (OBP)

*HSV-1 UL9 and EBV BZLF1 are both origin-binding proteins, but they share no homology in terms of sequence.

As far as cellular factors are concerned, several proteins interacting with some MHV68 vPIC components have now been identified (Lee et al., 2011). For example, TAX1BP1 (Tax1-binding protein) and PCBP1 [Poly(rC)-binding protein 1, also referred to as αCPs or hnRNP E] have been found to interact with ORF31 and ORF34, respectively, in a yeast two-hybrid screen. Modification of expression of either of these cellular proteins has significant – although opposite – effect on MHV-68 production. Whereas inhibiting TAX1BP1 expression reduces MHV-68 late gene expression, silencing PCBP1 increases it (Lee et al., 2011).

In the case of EBV, the cellular factor TSG101 (Tumor Susceptibility Gene 101) which is a component of the ESCRT1 (endosomal sorting complexes required for transport 1), implicated in different cellular functions including cytokinesis, protein ubiquitination, transcriptional regulation, cell cycle regulation and proliferation, as well as viral budding, was found to modulate the expression level of EBV late genes. In effect, upon virus reactivation, the level of several EBV late transcripts is markedly reduced in cells lacking TSG101. The underlying mechanism was found to be Rta-related (Chua et al., 2007).

Taken together, these studies strongly suggest that β- and γ-herpesviruses share a similar mechanism to regulate their late gene expression. The vPIC components that have been identified are probably functionally equivalent to the complex formed between some of the cellular PIC components and ICP4 in the case of α-herpesviruses.

## Link between Viral DNA Replication and vPIC Function

We have seen that the β- and γ-herpesvirus vPICs specifically stimulate the promoters of late viral genes. However, late gene expression not only depends on these virally encoded *trans*-acting factors but also on the accomplishment of viral DNA replication (Aubry et al., 2014). Several hypotheses have been proposed to explain this dependency: (i) DNA replication may stimulate late viral transcription because it increases the copy number of the viral genomes. Accordingly, some studies have shown that the increase in RNA transcription is proportional to the level of DNA amplification. This suggests that template amplification is a key determinant of the RNA transcription rate (Johnson et al., 1986). These results are consistent with a straightforward amplification model, whereby replication generates additional templates available for transcription; (ii) DNA amplification may allow titration of a putative repressor, and scanning of the DNA by the replication complex could alter the balance of positive- and negative-acting factors associated with the late viral promoters; (iii) proteins stably associated with the viral replication complex could have a dual role, one that is specific for the replication of the viral DNA, and a second by which they enhance transcription of viral late promoters when brought into their proximity; (iv) since DNA-replication occurs at nuclear sites localized near PML domains (Bell et al., 2000) it is possible that this location favors transcription from late promoters; (v) viral DNA replication may be important because it initiates the formation of replication factories in which late gene transcription would be favored; (vi) viral DNA replication may lead to the genesis of viral genomes with a structure different from that of non-replicated genomes that allows better access for the transcription machinery to the late gene promoters. The two last hypotheses appear at present to be the more realistic and will be developed further.

### Replication Factories

Cellular DNA replication occurs in microscopically visible structures (called replication factories) at discrete sites in the nucleus. These factories consist of DNA associated with the replication machinery that can be detected through their co-localization with the DNA clamp protein, PCNA (Proliferating Cell Nuclear Antigen). Thousands of these factories form in the nuclei during S phase (Leonhardt et al., 2000). They accumulate in early S phase, give rise to punctuate peri-nuclear structures in mid-S phase, and then start to decrease in number toward the end of S phase.

During latency, γ-herpesviruses are dependent on the cellular replication machinery and the replication of the viral episomes probably takes place in cellular replication factories. However, during the productive cycle, all herpesviruses amplify their DNA from a specific origin of replication – called OriS for HSV-1 and OriLyt for EBV (Hammerschmidt and Sugden, 1988) – using their own replication machinery. This viral DNA replication takes place in discrete sites in nuclei, called viral DNA replication compartments. With the progression of viral DNA replication, these compartments enlarge and fuse with one another to form large globular structures that eventually almost completely fill the nucleus at the later stages. Cellular chromatin is then tightly compacted and localized to the periphery of the nucleus (Monier et al., 2000; Chiu et al., 2013). It has recently been shown that even though the viral DNA replication complex mediates unlicensed replication of viral DNA, genome amplification is often delayed until the early S phase of the ensuing cell cycle. It is not clear how herpesvirus replication factories form, but similar to the cellular replication factories, they can be marked by PCNA staining, although the PCNA stays at the periphery of these structures (Daikoku et al., 2005; Chiu et al., 2013). Accordingly, PCNA is probably not required for viral DNA amplification since the virus encodes its own DNA clamp factor (BMRF1 in the case of EBV; Tsurumi et al., 1993). Analysis of the architecture of the viral replication compartments has revealed that BMRF1 is enriched in core structures which form specific domains inside the replication compartment (Sugimoto et al., 2011). BMRF1 forms a heterodimer with the EBV BALF5 polymerase catalytic subunit (Tsurumi et al., 1993) but also forms head-to-head homodimers or tetrameric ring structures (Murayama et al., 2009; Nakayama et al., 2010) that presumably contribute to the BMRF1-related core structures. The BMRF1 protein is also a polymerase processivity factor and may thus play a dual role during lytic replication: one assisting the viral polymerase during elongation and the other protecting the viral genome after synthesis. In effect, although *de novo* synthesis of viral DNA occurs mainly outside of the BMRF1 cores, replicated DNA is subsequently stored inside BMRF1 core structures (Sugimoto et al., 2011, 2013). Thus, viral DNA amplification and viral genome maturation appears to be, respectively, assigned to the outside and the inside of the BMRF1 cores. Using confocal laser scanning microscopy and 3D surface reconstruction imaging, Sugimoto et al have recently shown that viral early mRNAs are mainly found outside the BMRF1 cores whereas the viral late mRNAs are located inside (Sugimoto et al., 2013). In accordance with this observation, these authors found that the viral BcRF1 protein – the TBP-like EBV vPIC component – is localized inside BMRF1 cores, suggesting that viral late gene transcription takes place within these particular structures. These results indicate that there is a spatial relationship between the sites of viral late gene transcription and viral DNA replication.

Similarly, the UL87, UL95, and UL79 proteins from hCMV assemble with the viral genome into micro-foci containing the early viral protein UL44, which is structurally homologous to the EBV BMRF1 protein (Perng et al., 2011). As proposed for EBV, the recruitment of the UL44 protein together with UL79, UL87, and UL95, into the replication foci, provides an appropriate environment for late gene transcription. However, no direct interaction between UL44 and components of the hCMV vPIC has yet been described. This is also the case for the EBV proteins. Consequently, although it is still not known whether vPIC is recruited into the amplification core structure by the viral DNA replication machinery, a tight connection certainly exists between these two complexes.

### Viral Chromatin Modification

**FIGURE 5:**
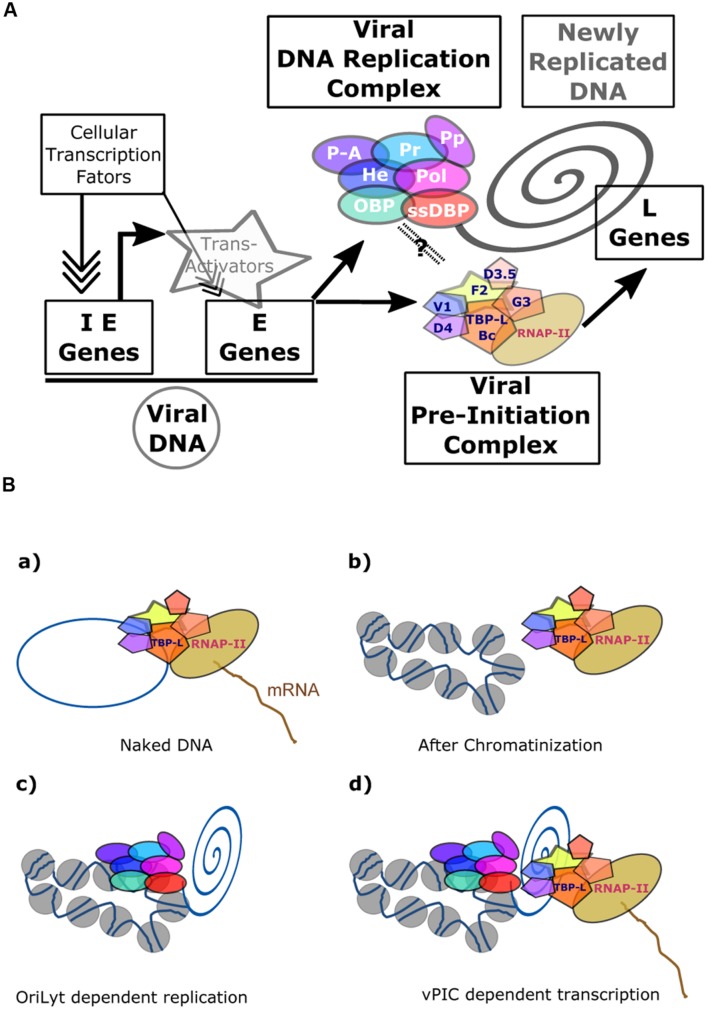
**Transcriptional regulation of β- and γ-herpesviruses late gene expression. (A)** Overview of EBV productive cycle. The viral immediate-early and early genes are transcribed from the episomic viral DNA and their expression controlled by the cellular transcription machinery formed around the cellular PIC. Expression of the late viral genes is dependent upon viral DNA replication and on the presence of the viral specific Pre-Initiation Complex (vPIC) composed of five viral proteins associated with the viral TATT-box binding protein like (TBP-L). **(B)** Expression of the late viral genes is linked to newly replicated DNA. vPIC alone can activate the expression of a late gene present on a plasmid during a transient transfection assay (a), but it is not able to induce the expression of a late viral gene present on a stable episome (b). The chromatinized DNA bearing the OriLyt origin of DNA replication can be replicated by the viral DNA replication complex (c). In these conditions, vPIC, present in the replication factories, can activate expression of the late viral genes (d). The link between the replication complex and vPIC is not proven but several arguments described in the main text strongly argue in favor of such a model. IE, immediate-early; E, early; L, late; OBP, origin-binding protein; He, helicase; Pol, polymerase; ssDBP, single-strand DNA-binding protein; Pr, primase: P-A, primase associated: PP, Polymerase processivity; TBP-L, TATA-binding protein like; Bc, BcRF1; F2, BFRF2; G3, BGLF3; V1, BVLF1; D4, BDLF4; D3.5, BDLF3.5.

The herpesvirus genome is maintained in host cells in an episomic state, covered by nucleosomes and carrying substantial epigenetic marks. Epigenetic regulation has been found to be important for viral gene expression (Cuevas-Bennett and Shenk, 2008; Bergbauer et al., 2010; Kalla et al., 2010; Chen et al., 2013; Lieberman, 2013). During the productive cycle, the viral replication complex amplifies viral DNA. As discussed above, the function of cellular PCNA – which is excluded from the replication factories (Chiu et al., 2013) – is not directly required for this. PCNA is known to recruit the cellular DNA methyl-transferase that performs CpG methylation of the newly synthesized DNA during the course of replication. Because of the exclusion of PCNA from the replication factories, cellular DNA methyl-transferase cannot be recruited onto the newly replicated DNA, leading to the genesis of viral genomic DNA deprived of methylation marks. This is in sharp contrast to the heavily methylated pattern associated with the viral genome during latency. However, it is important to note that treatment of cells by inhibitors of DNA methyl-transferase is not sufficient to induce synthesis of late EBV genes in Raji or HH514 cells (Szyf et al., 1985; Fronko et al., 1989). In addition to its interaction with the cellular DNA methyl-transferase, PCNA also interacts with the Chromatin Assembly Factor 1 (CAF-1). CAF-1 is a histone chaperone for the tetrameric formation and deposition of histones (H3-H4)_2_, and is found on replicating DNA directly behind the replicative helicase (Shibahara and Stillman, 1999). In the absence of PCNA, CAF-1 is not recruited onto replicated DNA and consequently, the newly synthesized viral DNA is not covered by histones. The newly synthesized viral DNA therefore appears to be devoid of chromatin structure in the replication factories. Finally, it has been shown that histones are excluded from the viral DNA replication foci during the productive cycle (Chiu et al., 2013). Such presumably “naked” DNA would be fully accessible to transcription factors provided they are present in the same structures (**Figure 5**). The hypothesis that viral late genes are transcribed by the vPIC from “naked” and unmethylated DNA is reinforced by the finding that vPIC alone is not able to induce expression of a late viral gene when it is present in an episome stably maintained in cells, whereas it can activate the expression of a late gene present on a plasmid during a transient transfection assay (Serio et al., 1997; Amon et al., 2004; Aubry et al., 2014).

## Conclusion/Future Directions

For a successful infection, DNA viruses require the coordinated expression of immediate-early, early and late genes. Immediate-early gene products are responsible for stimulating early gene expression, whose products are responsible for both viral DNA replication and late gene expression. Although not yet well understood, the mechanisms underlying late gene expression appear to differ greatly between various DNA viruses. In the case of simian virus 40, for example, the amplification of viral DNA is required *in trans* to attenuate the repression of viral late promoters (Wiley et al., 1993) and the viral large T antigen plays an essential role in activating the promoters (Keller and Alwine, 1984). In the case of adenoviruses, viral DNA replication facilitates late gene expression directly or indirectly by promoting the expression of viral *trans*-acting factors, or by the recruitment of cellular transcription factors to late promoters (Thomas and Mathews, 1980; Iftode and Flint, 2004; Morris and Leppard, 2009). For papillomaviruses, late gene expression is tightly associated with keratinocyte differentiation and is mediated in part by alternative mRNA splicing (Jia et al., 2009). The majority of eukaryotic DNA viruses, including α-herpesviruses, use a host-cell-derived transcription machinery to express their genes. However, some DNA viruses like baculoviruses or the T7 bacteriophage encode a viral-specific transcription complex, including a viral-encoded RNA-polymerase, necessary for expression of their late genes. The requirement for a specific viral RNA polymerase is probably due to the special structure of these late viral promoters. In effect, to promote late viral gene expression, viral RNA polymerases recognize unique promoter elements that are absent from cellular gene promoters. For instance, baculovirus RNA polymerase recognizes a TAAG element in the late and very late promoters, whereas T7 RNA polymerase exhibits a binding affinity to a 23-bp consensus sequence found on T7 bacteriophage late promoters. Among herpesviruses, α-herpesviruses have evolved differently to β- and γ-herpesviruses, in the mechanisms they use to express their late genes. Only β- and γ-herpesviruses encode a specific vPIC formed around a viral TBP-like protein recruited onto a TATT box motif that constitutes the only *cis*-regulatory element of their late gene promoters. The requirement for a specific viral TBP-like protein is important because cellular TBP is not very efficient in binding a TATT sequence. Accordingly, very few cellular gene promoters contain such a TATT-box. This is a marked similarity with baculoviruses and T7 bacteriophage for which late viral gene promoter structure differs from that of both early viral and cellular genes.

Although many aspects of herpesvirus replication and gene expression have recently been elucidated, the contribution of viral DNA replication to the process of viral late gene expression remains to be elucidated. Moreover, further studies will be needed to understand the role of each component of the β- and γ-herpesvirus vPIC in transcription initiation and elongation. Elucidation of the structure of the different vPIC components and extensive mutational analysis of these factors should facilitate the identification of their functional domains and mode of action. In addition, the comparative analysis of vPICs identified in different β- and γ-herpesviruses should help in the definition of the specific function of each vPIC component.

## Author Contributions

All authors listed, have made substantial, direct and intellectual contribution to the work, and approved it for publication.

## Conflict of Interest Statement

The authors declare that the research was conducted in the absence of any commercial or financial relationships that could be construed as a potential conflict of interest.
